# Influence of Artificial Intelligence and Robotics Awareness on Employee Creativity in the Hotel Industry

**DOI:** 10.3389/fpsyg.2022.834160

**Published:** 2022-03-01

**Authors:** Hui Wang, Han Zhang, Zhezhi Chen, Jian Zhu, Yue Zhang

**Affiliations:** Business School, Xiangtan University, Xiangtan, China

**Keywords:** artificial intelligence and robotics awareness, active learning, task crafting, locus of control, employee creativity, moderating multiple mediation model

## Abstract

The current literature in artificial intelligence and robotics awareness (AIRA) focused on the dark side of AIRA. Accordingly, this study sheds light on the positive effect of AIRA on employee creativity by exploring how and when hotel employees may take proactive behavior facing the threat of AI and robotics to further stimulate creativity. Based on the work adjustment theory (TWA) and the locus of control theory, this study constructs a moderating multiple mediation model to explain the influence of AIRA on employee creativity, in which active learning and task crafting are used as mediating variables, and locus of control is used as moderating variable. Data collected from 264 employees in a Chinese hotel are used for empirical analysis. Results show that (a) AIRA indirectly positively affects employee creativity *via* active learning and task crafting and (b) Locus of control not only moderates the mediating effect of active learning between the relationship of AIRA and employee creativity but also moderates the mediating effect of task crafting between the relationship of AIRA and employee creativity. The theoretical and practical implications are discussed.

## Introduction

The world is experiencing an era of remarkable changes caused by the advanced Fourth Industrial Revolution (Schwab, 2017). The advanced Fourth Industrial Revolution affects our life, work, and production through artificial intelligence (AI), robotics, automation, and digitization (Ivanov and Webster, 2019; Hwang et al., 2020), which are seen as the breakthroughs of emerging technology. These technologies, especially AI and robotics, have exhibited profound effects on a large number of sectors such as the hotel industry (Blöcher and Alt, 2020; Henkel et al., 2020; Tussyadiah, 2020). AI and robotics are widely used in hotels worldwide which can replace some repetitive and tedious tasks. The hotels in the United States, such as The Wynn Las Vegas and Aloft, have already installed virtual assistants that respond to guest requests in rooms (Li et al., 2019). Japan has a robot hotel, called Henn na Hotel, where exist robot porters, a cloakroom robot, and in-room personal assistants (Tussyadiah, 2020). In China, some service robots are also adopted in hotels. When using AI and robots in hotels becomes a tendency, the impact of AI and robotics on hotel employees who face the risk of being replaced by AI and robots is worth considering.

Some scholars began to focus on employees’ responses to AI and robotics (Thompson, 2005; Grant et al., 2010). Li et al. (2019) created artificial intelligence and robotics awareness (AIRA) of employees in the hotel industry, which means the extent to which employees have the feeling of replacement by AI and robotics. At present, the literature scarcely focuses on the outcomes of AIRA. Limited studies also exist on AIRA that focuses on the negative outcomes brought by AIRA, including leading to turnover intention (Li et al., 2019) and being harmful to employees’ mental health (Brougham and Haar, 2017). However, research on the bright side of AIRA has been scarce, especially on the positive influence of AIRA on employee creativity. Creativity, which refers to the production of novel and useful ideas on products, services, or procedures (Amabile, 1988; Carmeli, 2009), has become increasingly vital for an enterprise to succeed in the competitive business environment (Jaiswal and Dhar, 2017).

To address this theoretical gap, this study constructs a theoretical model to test the influence of AIRA on employee creativity. As the work adjustment theory (TWA) proposed, individuals will determine ways to seek and maintain person-environment fit (Dawis and Lofquist, 1976). When an employee has AIRA, they will worry about the changes in the work environment brought by the usage of AI and robotics, which may interrupt the person-environment fit. Thus, employees who have AIRA will take proactive behaviors to achieve person-environment fit. One proactive behavior is active learning, which refers to employees actively controlling their learning process. Active learning helps employees gain knowledge and skills to cope with the challenge of AI and robotics (Taris et al., 2003; de Jonge et al., 2012). Another proactive behavior is task crafting, which means employees change the extent or amount of tasks in their job to better fit their skills and interests (Tims et al., 2012; Slemp and Vella-Brodrick, 2013; Rudolph et al., 2017). Task crafting enables employees to reshape their tasks and cooperate better with AI and robotics. These two proactive behaviors concentrate on different aspects of reaching person-environment fit. Furthermore, both active learning and task crafting are beneficial for employees generating creative ideas (Berg et al., 2010). Consequently, this study constructs a parallel dual mediation model to explore the influence of AIRA on employee creativity with active learning and task crafting as mediators.

Moreover, the locus of control theory holds that locus of control is a kind of personal disposition that people attribute the reason why things happened to either internal or external sources, individuals with an internal locus of control believe that they have the strength to overcome the threat, whereas individuals with an external locus of control perceive themselves powerless and attribute the reason for success or failure to the external environment (Spector, 1988; Allen et al., 2005; Aubé et al., 2007; Chen and Silverthorne, 2008). According to the locus of control theory, compared with individuals with an external locus of control, individuals with an internal locus of control will more positively take proactive behaviors to cope with AIRA. Thus, this study considers locus of control as a moderator to explore the boundary conditions of when AIRA affects employee creativity *via* active learning and task crafting.

Overall, this study integrates TWA and the locus of control theory to construct a moderating multiple mediation model of AIRA affecting employee creativity, which uses active learning and task crafting as mediating variables and locus of control as a moderating variable. This study tends to shed light on the positive effect of AIRA on employee creativity by exploring how and when a hotel employee may take proactive behavior facing the threat of AI and robotics to further stimulate creativity. Thus, this study has the following contributions. First, this study broadens the relevant studies on AIRA. At present, limited attention has been paid to AIRA, and previous studies concentrated on the negative effect of AIRA. This study explores the positive effect of AIRA on employee creativity. Second, this study further explores how AIRA affects employee creativity. Based on TWA, this study proposes that AIRA indirectly affects employee creativity *via* active learning and task crafting. Third, this study explores the boundary conditions of the influence of AIRA on employee creativity. Employees with an internal locus of control will believe in themselves more than employees with an external locus of control. Therefore, employees with an internal locus of control will have a stronger link between AIRA and active learning and task crafting, which further leads to employee creativity.

### Theoretical Background and Hypotheses

#### Artificial Intelligence and Robotics Awareness

Both AI and robotics have received considerable attention from researchers and business managers since they have been used in service sectors (Syam and Sharma, 2018; Huang and Rust, 2018; Henkel et al., 2020; Mingotto et al., 2020; Salas-Pilco, 2020; Tussyadiah, 2020). At present, the literature substantially focuses on the negative effect of AI and robotics usage on employees. Most of the studies indicate that employees, especially those in low-skilled positions, are facing a high risk of displacement by AI and robotics (Autor and Dorn, 2013; Frey and Osborne, 2017; Huang and Rust, 2018; Manthiou et al., 2020). Considering the negative effects of AI and robotics, exploring how employees cope with such a crisis is essential. Li et al. (2019) created a concept of AIRA to capture the feeling of employees on AI and robotics in the hotel sector. AIRA is a subjective sense of how employee considers the threat of AI and robotics, which is not exactly the same as objective AI and robotics. Limited studies on AIRA focus on the negative outcomes brought by AIRA. For example, Li et al. (2019) suggested that AIRA may lead to employee turnover intention because the anxiety of displacement will motivate employees to choose another job. Brougham and Haar (2017) argued that AIRA will be negatively associated with employee psychological health. Considering that employees worry about the risk of being fired in the future, they inevitably have a feeling of anxiety, stress, or burnout. However, no literature exists on the influence of AIRA on employee creativity, thus far. Similar to a Chinese saying, “Born in misery, died in peace,” the new changes brought by AI and robotics may be a warning to encourage improvement. Although employees are at risk of replacement, it is also an opportunity for them to take proactive actions to achieve person-environment fit (Manthiou et al., 2020), which will further lead to positive outcomes (Caldwell and Iii, 1990; Lee et al., 2021).

#### Employee Creativity

Creativity is defined as the production of novel and useful ideas on products, services, or procedures (Amabile, 1988; Carmeli, 2009). Amabile et al. (1996) further developed a componential creativity framework, which includes three components, namely, domain-relevant skills, motivation, and creativity-relevant processes, and they proposed that an individual can be more creative when these three components share their best combinations. The hotel industry is a customer-oriented service industry involving frequent interactions between customers and employees. Consequently, the creativity of an employee in the hotel industry refers to his/her ability to generate creative ideas to improve customer service quality and enhance customer satisfaction (Li et al., 2018; Ruiz-Palomino and Zoghbi-Manrique-de-Lara, 2020; Chien et al., 2021). Employee creativity is vital for an enterprise to gain success in the competitive hotel industry. Previous research on employee creativity focused on the impact of individual factors or external factors, such as personalities, cognitive process, or leadership (Barron and Harrington, 1981; Schooler and Melcher, 1995; Liu et al., 2012). As research further developed, scholars began to concentrate on the interaction between individual factors and contextual ones. The correspondence between individuals and the external environment can promote creativity (Livingstone et al., 1997; Wang and Wang, 2018).

#### The Mediating Role of Active Learning on the Relationship Between Artificial Intelligence and Robotics Awareness and Employee Creativity

Active learning is often mentioned in the education field but gradually adopted to express the proactive action of employee learning in the work context in recent years (Taris et al., 2003; Katz-Navon et al., 2009; de Jonge et al., 2012; Bergh et al., 2013). Active learning behavior in the context of work is known as employee development (Simmering et al., 2003) and refers to self-initiated, self-directed behavior by means of which employees improve their competencies to fit the change in the work environment. Active learning has three characteristic components. First, active learning implies that employees have a motivation to learn whereby they start learning activities themselves (Simmering et al., 2003; Taris et al., 2003). Second, active learning means that employees can have control over the learning process (Bell and Kozlowski, 2008). Third, employees involved in active learning experience a feeling of high self-efficacy (Taris et al., 2003).

Drawing on TWA, an individual’s behavior is influenced by the relationship between individual and external environments (Dawis and Lofquist, 1984). When employees possess knowledge and skills which fit with their job, they tend to behave actively. The usage of AI and robotics in the hotel industry prompts the change in the work environment, which may further cause employees’ awareness of mismatch in their job. Consequently, employees who have AIRA may take proactive actions to fit themselves in the changing work environment. Following this perspective, AIRA will lead to active learning (Taris et al., 2003), which then helps employees gain skills and knowledge (Huang and Rust, 2018) to enhance their creativity.

On the one hand, AIRA can positively predict employees’ active learning. Active learning means that employees are motivated to learn, whereby they start learning activities themselves (Simmering et al., 2003; Taris et al., 2003). As prior studies suggested, AI and robotics will replace employees in low-skilled jobs (Wirtz and Jerger, 2017), and the people who own soft or non-routine skills, such as communicating, listening, and negotiating skills, are impossibly replaced by robot service in the present time (Metzler et al., 2016). Therefore, AIRA forces employees in the hotel industry to engage in active learning to enrich their knowledge and improve their skills (Huang and Rust, 2018), which enables them to become more competitive and more irreplaceable. Active learning is the exact way taken by employees for acquiring new knowledge and skills. Thus, AIRA will have a positive influence on active learning.

On the other hand, active learning is positively related to employee creativity. Creativity consists of the generation of ideas, products, or services judged as novel and useful by external observers (Woodman et al., 1993; Amabile et al., 1996). This quality requires the application of existing knowledge and the development of appropriate new knowledge (Gurteen, 1998). Furthermore, individuals with broad knowledge have more flexible knowledge structures thanks to their exposure to different domains; in doing so, individuals have a greater ability to recombine knowledge across different domains to generate creative ideas (Perry-Smith and Shalley, 2003; Taylor and Greve, 2006; Sosa, 2011; Mannucci and Yong, 2018). Active learning contributes to accumulating employees’ knowledge and improving employees’ skills. An active learner can obtain more knowledge and skills, making available the information and knowledge that facilitates employee creativity. Previous research on the outcomes of active learning has suggested that active learning can reduce strain, reach high levels of productivity, and gain innovative ideas (Amabile, 1988; Karasek and Theorell, 1990; Taris et al., 2003). Therefore, this study proposes that AIRA will be positively related to employee creativity through active learning.

*Hypothesis 1a: AIRA is positively related to active learning.*
*Hypothesis 1b: Active learning is positively related to employee creativity.*
*Hypothesis 1c: Active learning mediates the positive association between AIRA and employee creativity.*

#### The Mediating Role of Task Crafting on the Relationship Between Artificial Intelligence and Robotics Awareness and Employee Creativity

Job crafting is defined as a self-initiated behavior that employees take to change the features of their job in physical, cognitive, and social aspects (Slemp and Vella-Brodrick, 2013). Job crafting is a sort of proactive behavior which includes task crafting, cognitive crafting, and relational crafting (Wrzesniewski and Dutton, 2001; Grant and Ashford, 2008; Petrou et al., 2012). Task crafting means that employees change the number, scope, and type of tasks in their job to better fit their skills. Cognitive crafting involves changing employees’ views of their jobs to make their job more meaningful. Relational crafting refers to the reshaping of relationships in employees’ jobs. Compared with task crafting, cognitive crafting and relational crafting are indirect ways to reshape their job, which may not be the primary ways to cope with the threat (Lin et al., 2017). Therefore, this study emphasizes on task crafting.

As TWA proposed, the fit between individual and work environment can be achieved in two ways: individuals change themselves to be accepted by the environment, and the environment is changed to be acceptable for the individual (Dawis and Lofquist, 1984). Active learning emphasizes that employees strengthen themselves to better fit their job, while task crafting emphasizes that employees reshape tasks to help them better fit their job (Tims et al., 2016). The usage of AI and robotics in the hotel industry prompts the change of work environment, which may further cause employees’ awareness of mismatch in their job. Therefore, AIRA will lead to employees’ task crafting, which enables employees to reshape their tasks and cooperate better with AI and robotics, and in turn benefit employee creativity.

On the one hand, AIRA may lead to task crafting. As Huang and Rust (2018) proposed, the AI and robotics replacement will first happen in the task. When employees have AIRA, they probably take direct actions in their tasks to overcome such a threat. Facing the probability of losing their jobs, employees tend to reshape their tasks to reduce conflict with AI and robotics, thus enabling them to win back their initiative in work. Just as Huang and Rust (2018) assertion, the cooperation between employees and AI and robotics should be the focus of management. Task crafting helps employees work rather than fight against robotics. Therefore, AIRA is positively associated with task crafting.

On the other hand, during the process of task crafting, employees will work autonomously and redesign the task content according to the allocation of various resources (Zeijen et al., 2018). Accordingly, resources will collide with each other in the redistribution of resources, which will be conducive to the emergence of new ideas and thus generate creativity (Wang and Lau, 2021). Furthermore, task crafting is a process in which employees generate various ideas and attempt to view their tasks from unconventional perspectives (Slemp and Vella-Brodrick, 2013), which inspires creative thinking and further leads to creativity. Consequently, task crafting is positively related to employee creativity. This study further proposes that AIRA indirectly affects employee creativity through task crafting.

*Hypothesis 2a: AIRA is positively related to task crafting.*
*Hypothesis 2b: Task crafting is positively related to employee creativity.*
*Hypothesis 2c: AIRA indirectly affects employee creativity through task crafting.*

#### The Moderating Role of Locus of Control

According to the locus of control theory, locus of control describes a type of personality that people tend to attribute the reason why things happened to either internal sources or external sources (Anderson, 1977; Ajzen, 2002; Allen et al., 2005; Chen and Silverthorne, 2008). Employees with an internal locus of control have faith in their own ability to control things that happened to them. They take responsibility for their success and failure. Conversely, employees with an external locus of control attribute the causes of success and failure to external factors beyond their control. Individuals with different kinds of locus of control tend to take different responses to challenges. Employees with an internal locus of control believe that they can control negative threats (Glass and Singer, 1973), and they choose to take more active ways to cope with them (Tims et al., 2012). Additionally, employees with an internal locus of control will choose task-focused coping behavior instead of emotion-focused coping behavior (Anderson, 1977).

Drawing on the locus of control theory, when facing the risk of replacement caused by AI and robotics, employees with an internal locus of control believe that they have the power to rise to the challenge. Thus, they will take proactive actions to reduce the negative impact of using AI and robotics. In contrast, employees with an external locus of control tend to be powerless and take less proactive actions to fight against difficulties. Thus, this study proposed that locus of control moderates the relationship between AIRA and proactive behaviors which refer to active learning and task crafting.

*Hypothesis 3a: Locus of control moderates the positive relationship between AIRA and active learning. The relationship is stronger when employees tend to show internal locus of control.*
*Hypothesis 3b: Locus of control moderates the positive relationship between AIRA and task crafting. The relationship is stronger when employees tend to show internal locus of control.*

Furthermore, based on the above discussion, locus of control acts as a moderator between AI and robotics awareness and proactive behaviors (active learning and task crafting). Then, proactive behaviors in turn predict employee creativity. Anderson (1977) indicated, under negative conditions, employees with an external locus of control are more likely to feel stress and anxiety. Employees with an internal locus of control are more willing to take proactive actions to address problems, which predicts further success (Tims et al., 2012). In the process of overcoming challenges by taking proactive actions, internal individuals will have more possibility to show creativity than external ones.

*Hypothesis 4a: Locus of control moderates the indirect positive effect of AIRA on employee creativity via active learning. The indirect positive effect is stronger when employees tend to show internal locus of control.*
*Hypothesis 4b: Locus of control moderates the indirect positive effect of AIRA on employee creativity via task crafting. The indirect positive effect is stronger when employees tend to show internal locus of control.*

**FIGURE 1:**
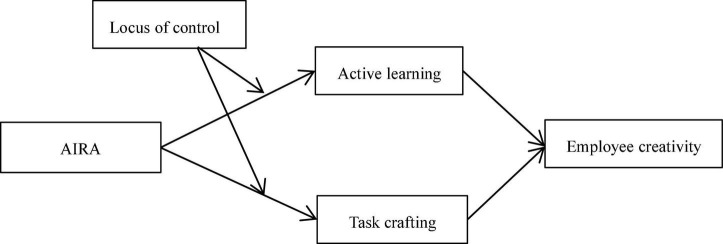
Theoretical model.

To conclude, Figure 1 shows the hypothetical model of this study.

## Materials and Methods

### Sample and Procedure

To test the abovementioned hypotheses, we collected data from employees working in the hotel industry in China. We chose China’s hotel industry to conduct our empirical research for two reasons. First, AI and robotics which serve as an assistant in Chinese hotels become common, and the hotel industry provides extensive application scenarios, which helps us obtain sufficient information to continue our study. Second, as Autor and Dorn (2013) suggested, AI and robotics will first become a substitute for low-wage service occupations. Thus, the effect of AI and robotics on service employees is larger than in other occupations. The hotel industry can provide a large body of samples of service workers for our study.

This study employed the snowball sampling approach to construct the company sample (Hendricks and Blanken, 1992). First, 20 hotels in China are identified through the MBA alumni. Second, the human resource department directors of these hotels were contacted, and the purpose of data collection was explained. From these 20 hotels, 315 employees were recruited to participate in the questionnaire survey. A private email was sent to all participants several days before the questionnaire survey to explain the research procedure and to emphasize that the survey is for academic research purposes only and strictly under complete confidentiality. Then, the questionnaire link was emailed to 315 participants.

Collecting data in different waves for the independent and dependent constructs can benefit from migrating common method bias (Podsakoff et al., 2012). Accordingly, two data collection waves were implemented. In Time 1 (T1), employees complete questionnaires regarding independent variable (AIRA), moderating variable (locus of control), and demographic variables (age, gender, education, department, and position). After a month, at Time 2 (T2), the same participants completed questionnaires regarding mediating variables (active learning and task crafting) and a dependent variable (employee creativity).

A total of 51 questionnaires were discarded for missing data or patterned responses, such as alternating between options or clicking the midpoint, or random responses (Mckibben and Silvia, 2015), leaving 264 valid questionnaires and a response rate at 83.8%. Table 1 shows the sample description.

**TABLE 1:** Sample description.

Variable	Feature	Frequency	Ratio (%)	Variable	Feature	Frequency	Ratio (%)
Gender	Male	75	28.40	Department	Room service	37	14
	Female	189	71.60		Food and beverage service	81	30.70
Age	20 years old and below	36	13.60		Logistics service	6	2.30
	21–30 years old	126	47.70		Front office service	50	18.90
	31–39 years old	35	13.30		Marketing	19	7.20
	40 years old and over	67	25.40		Administration	20	7.60
Education	High school degree and below	78	29.50		Human resource	15	5.70
	Junior college degree	64	24.20		Accounting	12	4.50
	Bachelor degree	115	43.60		Others	24	9.10
	Master degree and over	7	2.70	Position	Senior manager	18	6.80
Length of working in present hotel	Under 3 years	159	60.20		Middle manager	45	17
	3–5 years	40	15.20		Junior manager	39	14.80
	6–9 years	38	14.40		General staff	162	61.40
	Over 10 years	27	10.20				

### Measures

All scales were adopted from the literature in English and were translated into Chinese in this study for data collected from Chinese employees. To ensure the accuracy of the translation, we followed the standard translation and back-translation procedures (Brislin, 1970). All measures were rated from 1 (strongly disagree) to 5 (strongly agree). AIRA is measured with a four-item scale developed by Li et al. (2019), active learning is measured with a four-item scale developed by Taris et al. (2003), task crafting is measured with a five-item scale developed by Slemp and Vella-Brodrick (2013), employee creativity is measured with a four-item scale developed by Slemp and Vella-Brodrick (2013), and locus of control is measured with an eight-item scale developed by Farmer et al. (2003). Table 2 presents the detailed items of those scales. In addition, following previous research (Hon et al., 2013; Tongchaiprasit and Ariyabuddhiphongs, 2016; Wang, 2016) and our study context, this study selected several control variables including gender, age, education, length of working in the present hotel, department in the present hotel, and position in the present hotel.

**TABLE 2:** Items for measurement.

Variable	Item No.	Item content
AIRA	AIRA1	I am worried that my work will be replaced by artificial intelligence machine
	AIRA2	I am worried that what I do now in my job may be replaced by machines with AI and robotics
	AIRA3	I am very pessimistic about the future of the hotel where I work, because employees may be replaced by AI systems
	AIRA4	I am pessimistic about the future of the hotel industry as a whole, because employees may be replaced by AI systems
AL	AL1	I am constantly looking for new challenges in my work
	AL2	I try hard to keep up with the latest developments
	AL3	When things don’t seem right, I try harder and try again
	AL4	At work, I spend time learning new things
TC	TC1	I will introduce new ideas to improve my work
	TC2	I will change the scope or type of tasks in my job
	TC3	I bring in new tasks that better match my skills or interests
	TC4	I choose to take on extra tasks at work
	TC5	I prioritize task that matches my skills or interests
EC	EC1	I’m always inclined to try new ideas or approaches
	EC2	I try to find new ideas and new ways to solve problems
	EC3	I can generate breakthrough ideas related to the field
	EC4	I’m a creative person
LOC	LOC1	Work is what you make it
	LOC2	In most jobs, individuals can accomplish almost anything they set out to do
	LOC3	If I knew what I wanted from a job, I could find a job that would give me those things
	LOC4	I think employees should take the initiative to make changes if they are not satisfied with their leader’s decision
	LOC5	Most people can do their jobs well as long as they work hard
	LOC6	Promotions are offered to employees who perform well
	LOC7	Rewards are offered to employees who perform well
	LOC8	Most employees have more influence over their leaders than they think

*AL, active learning; LOC, locus of control; TC, task crafting; EC, employee creativity.*

### Data Analysis

This study used SPSS 25.0 and Process v3.5 to analyze the data. First, SPSS 25.0 was used to test the reliability of five core variables in the theoretical model, and both descriptive statistics and correlation analysis were conducted. Second, Mplus 7.4 was used to conduct confirmatory factor analysis (CFA), which helps the further analysis of average variance extracted (AVE), convergent validity, and discriminant validity. Third, Process v3.5, a plug-in in SPSS 25.0, was used to examine the theoretical model. The multiple mediation model was tested in model 4 of Process v3.5 with the bootstrapping method. The moderating model was tested in model 1 of Process v3.5, and we applied mean center for continuous variables that define products to reduce potential collinearity. Moreover, the moderating multiple mediation model was tested in model 7 of Process v3.5 with the bootstrapping method.

## Results

### Reliability and Validity Test

To test the reliability, SPSS 23.0 was used to calculate Cronbach’s alpha, and the results show that Cronbach’s alpha of AIRA, active learning, task crafting, locus of control, and employee creativity were 0.810, 0.835, 0.891, 0.891, and 0.820, respectively. Cronbach’s alpha values of all variables were greater than 0.7, indicating that the questionnaire had good reliability. The AVE of AIRA, active learning, task crafting, locus of control, and employee creativity were 0.518, 0.529, 0.505, 0.512, and 0.540, respectively. The AVE values of all variables were greater than the critical standard of 0.5 and squared correlations between variables, which demonstrated good convergent validity and discriminant validity. Furthermore, Mplus 7.4 was used to carry out CFA. The hypothetical five-factor model (i.e., AIRI, active learning, task crafting, locus of control, and employee creativity) had a better fit to the data (χ^2^/df = 1.596, RMESA = 0.048, TLI = 0.942, CFI = 0.949, SRMR = 0.039) than the other competition models (Table 3). The results of CFA further confirmed discriminant validity.

**TABLE 3:** Results of confirmatory factor analysis (CFA).

	Factors	χ^2^	df	χ^2^/df	RMESA	TLI	CFI	SRMR
Five-factor model	AIRA,AL,LOC,TC,EC	423.055	265	1.596	0.048	0.942	0.949	0.039
Four-factor model	AIRA, AL + LOC, TC, EC	655.329	269	2.436	0.074	0.860	0.875	0.065
Three-factor model	AIRA, AL + LOC + TC, EC	783.255	272	2.880	0.084	0.817	0.834	0.066
Two-factor model	AIRA + AL + LOC + TC, EC	1090.795	274	3.981	0.106	0.710	0.735	0.090
One-factor model	AIRA + AL + LOC + TC + EC	1144.271	275	4.161	0.109	0.692	0.718	0.091

*AL, active learning; LOC, locus of control; TC, task crafting; EC, employee creativity.*

### Common Method Variance

As all variables in this study were measured by employee’s self-evaluation, the problem of common method variance needs consideration. Harman’s single-factor test was used to determine the degree of common method variance. From principal component factor analysis without rotation in SPSS 25.0, the results showed that the first principal component has explained 37.5% loading (< 50%), which referred to the absence of serious common method variance (Woszczynski and Whitman, 2004).

### Descriptive Statistics and Correlation Analysis

Table 4 shows the results of descriptive statistics (mean, SD) and correlation analysis (Pearson’s correlation coefficient). AIRA is positively correlated with active learning (*r* = 0.227, *p* < 0.01), task crafting (*r* = 0.244, *p* < 0.01), and employee creativity (*r* = 0.279, *p* < 0.01). Employee creativity is positively correlated with active learning (*r* = 0.637, *p* < 0.01) and task crafting (*r* = 0.674, *p* < 0.01). Locus of control is positively correlated with active learning (*r* = 0.545, *p* < 0.01) and task crafting (*r* = 0.572, *p* < 0.01). The correlations among core variables provide initial support for the further test of our theoretical model.

**TABLE 4:** Means, SD, and correlation.

	AIRA	AL	TC	EC	LOC	Gender	Age	Edu	Low	Depart	Post
AIRA	1										
AL	0.227**	1									
TC	0.244**	0.686**	1								
EC	0.279**	0.637**	0.674**	1							
LOC	0.158*	0.545**	0.572**	0.602**	1						
Gender	−0.035	0.052	0.094	0.012	0.097	1					
Age	−0.058	0.118	−0.017	−0.055	−0.077	−0.060	1				
Edu	0.092	−0.051	0.072	0.038	0.001	−0.024	−0.429**	1			
Low	0.086	0.158*	0.037	0.049	0.016	−0.065	0.489**	−0.138*	1		
Depart	−0.134*	0.099	−0.001	0.093	0.055	−0.219**	0.225**	0.051	0.178**	1	
Post	−0.068	−0.205**	−0.114	−0.183**	−0.097	0.154*	−0.243**	−0.089	−0.441**	−0.389**	1
Mean	3.379	3.924	4.013	3.957	3.719	1.72	2.50	2.19	1.75	3.97	3.31
SD	0.680	0.573	0.515	0.570	0.590	0.452	1.017	0.896	1.046	2.534	0.983

N = 264. SD, standard deviation; AL, active learning; LOC, locus of control; TC, task crafting; EC, employee creativity; Edu, Education; Low, length of working in present hotel; Depart, department in present hotel; Post, position in present hotel. **p < 0.01, *p < 0.05.

### Hypotheses Testing

First, Process v3.5 was used to test the multiple mediator models by using the bootstrapping method. The sample size of bootstrapping is 5,000, and 95% CI is involved in the results. Tables 5, 6 show the results. As shown in Table 5, AIRA is positively related to active learning (β = 0.199, *p* < 0.001, M1) and task crafting (β = 0.183, *p* < 0.001, M2), which supports Hypotheses 1a and 2a. Active learning is positively related to employee creativity (β = 0.303, *p* < 0.001, M3), and task crafting is positively related to employee creativity (β = 0.482, *p* < 0.001, M3). Therefore, Hypotheses 1b and 2b are supported. As shown in Table 6, the indirect effect of AIRA on employee creativity *via* active learning is 0.061, which is significant [95% CI = (0.024, 0.108), excluding 0]. It demonstrates the positive mediating effect of active learning. Meanwhile, the indirect effect of AIRA on employee creativity *via* task crafting is 0.088, which is significant [95% CI = (0.038, 0.140), excluding 0]. It confirms the positive mediating effect of task crafting. Thus, Hypotheses 1c and 2c are supported.

**TABLE 5:** Results of direct effect test.

Predictors	AL (M1)			TC (M2)			EC (M3)		
			
Independent variables	Effect	SE	*t*	Effect	SE	*t*	Effect	SE	*t*
AIRA	0.199***	0.051	3.891	0.183**	0.047	3.916	0.089*	0.038	2.342
Mediators									
AL							0.304***	0.060	5.030
TC							0.482***	0.066	7.277
*R* ^2^	0.113			0.086			0.546		
F	4.076***			2.997***			30.433***		

AL, active learning; LOC, locus of control; TC, task crafting; EC, employee creativity. *** p < 0.001, ** p < 0.01,*p < 0.05. The control variables are not shown in the sheet for brevity. The effects of results are non-standardized.

**TABLE 6:** Results of mediating effect test.

	Effect	Boot SE	Boot LLCI	Boot ULCI
Total indirect	0.149	0.037	0.075	0.218
AIRA → AL → EC	0.061	0.021	0.024	0.108
AIRA → TC → EC	0.088	0.026	0.038	0.140

*AL, active learning; LOC, locus of control; TC, task crafting; EC, employee creativity.*

*The effects of results are non-standardized.*

**FIGURE 2:**
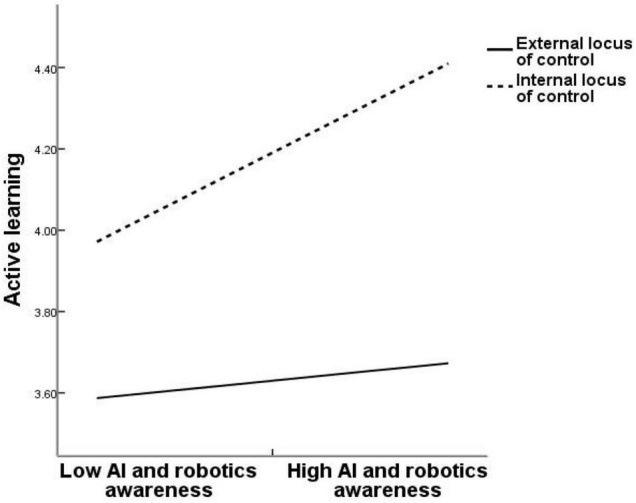
Interaction effect of locus of control and artificial intelligence and robotics awareness (AIRA) on active learning.

**FIGURE 3:**
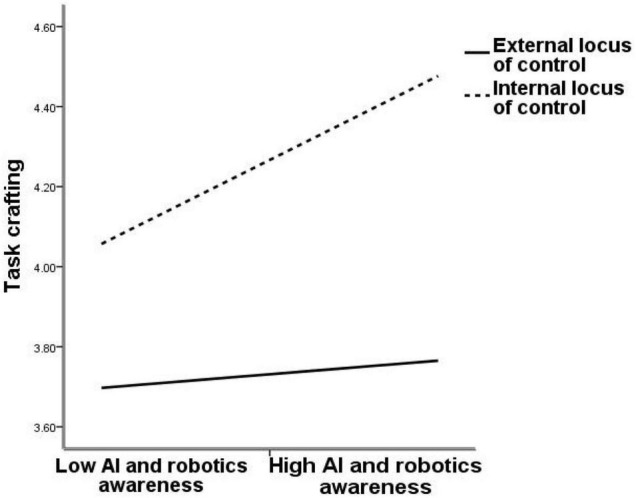
Interaction effect of locus of control and AIRA on task crafting.

Second, Process v3.5 was used to test the moderated effects of the locus of control. Table 7 shows the result. The interactive effect of AIRA and locus of control on active learning is significant (β = 0.22, *p* < 0.001, M4), which indicates that the locus of control positively moderates the relationship between AIRA and active learning. Furthermore, Figure 2 demonstrates that with internal locus of control (1 SD above the mean), AIRA is more positively related to active learning (β = 0.322, *p* < 0.001) than external locus of control (1 SD below the mean; β = 0.063, *p* > 0.05). Therefore, Hypothesis 3a is confirmed. Meanwhile, the interactive effect of AIRA and locus of control on task crafting is significant (β = 0.219, *p* < 0.001, M5). The results show that the locus of control positively moderates the relationship between AIRA and task crafting. Figure 3 demonstrates that with internal locus of control (1 SD above the mean), AIRA is more positively related to task crafting (β = 0.309, *p* < 0.001) than external locus of control (1 SD below the mean; β = 0.050, *p* > 0.05). Thus, Hypothesis 3b is supported.

**TABLE 7:** Results of moderating effect test.

variables	AL (M4)			TC (M5)		
		
	Effect	SE	*t*	Effect	SE	*t*
AIRA	0.193***	0.046	4.147	0.179***	0.0412	4.346
LOC	0.475***	0.05	9.571	0.454***	0.044	10.288
AI × LOC	0.220***	0.058	3.790	0.219***	0.051	4.260
*R* ^2^	0.394			0.408		
ΔR^2^	0.034***			0.043***		
*F*	16.443***			17.408***		

*AL, active learning; LOC, locus of control; TC, task crafting; EC, employee creativity.*

SE, standard error. ***p < 0.001. The control variables are not shown in the sheet for brevity. The effects of results are non-standardized.

Third, Table 8 shows the results of the moderated mediating effect test. Path 1 in Table 8 shows that in the group of internal locus of control, the indirect effect of AIRA on employee creativity *via* active learning is 0.098, which is significant [95% CI = (0.04, 0.169), excluding 0], whereas, in the group of external locus of control, the indirect effect of AIRA on employee creativity *via* active learning is 0.019, which is insignificant [95% CI = (−0.009, 0.054), containing 0]. In addition, the index of moderated mediation is 0.067 [95% CI = (0.020, 0.124), excluding 0]. Thus, the indirect effect of AIRA on employee creativity *via* active learning is significantly moderated by locus of control, and Hypothesis 4a is supported. Meanwhile, path 2 in Table 8 demonstrates that in the group of internal locus of control, the indirect effect of AIRA on employee creativity *via* task crafting is 0.149, which is significant [95% CI = (0.076, 0.228), excluding 0], whereas, in the group of external locus of control, the indirect effect of AIRA on employee creativity *via* task crafting is 0.024, which is significant [95% CI = (−0.024, 0.067), excluding 0]. Moreover, the index of moderated mediation is 0.106 [95% CI = (0.037, 0.182), excluding 0]. Therefore, the indirect effect of AIRA on employee creativity *via* task crafting is significantly moderated by locus of control; thus, Hypothesis 4b is confirmed.

**TABLE 8:** Results of moderated mediating effect test.

	Effect	Boot S.E.	Boot LLCI	Boot ULCI
**Path 1: AIRA → active learning → employee creativity**				
External locus of control (−1 SD)	0.019	0.016	−0.009	0.054
Internal locus of control (+ 1 SD)	0.098	0.033	0.040	0.169
Index of moderated mediation	0.067	0.027	0.020	0.124
**Path 2: AIRA → task crafting → employee creativity**				
External locus of control (−1 SD)	0.024	0.023	−0.024	0.067
Internal locus of control (+ 1 SD)	0.149	0.039	0.076	0.228
Index of moderated mediation	0.106	0.037	0.037	0.182

*AL, active learning; LOC, locus of control; TC, task crafting; EC, employee creativity.*

*The effects of results are non-standardized.*

## Discussion

On the bases of TWA and locus of control theory, this study constructs a moderating multiple mediation model to explore how and when AIRA exerted positive effect on employee creativity. Consistent with the hypotheses, the theory model is confirmed by empirical research. The conclusions are as follows:

First, AIRA indirectly posed positive effect on employee creativity through active learning and task crafting. As AI and robotics may bring threat to employees (Frey and Osborne, 2017; Huang and Rust, 2018), employees can take proactive behavior to cope with it instead of escaping it. This study mentioned two types of proactive behavior. Active learning helps employee gain new knowledge and skills, which enable them to become more competitive. Additionally, task crafting provides employees a way to reshape their job in the content and amount, which helps them better fit their job and cooperate with AI and robotics. In addition, the active learning and task crafting taken by employees will lead to employee creativity. By contrast, employees who have AIRA may make effort to active learning, so as to enrich their knowledge and improve their skills, which enables them to become more irreplaceable,. In turn the accumulated knowledge and improved skills benefit to employees creativity. Meanwhile, employees who have AIRA probably take task crafting to overcome the threat of replacement by AI and robots, and the process of task crafting will stimulates creative ideas which improves employee creativity.

Second, the indirect effect of active learning and task crafting are moderated by locus of control. Individual’s behavior may vary from person to person. Thus, this study considers the moderated effect of personality on the relationship between AIRA on employee creativity *via* active learning and task crafting. Drawing on locus of control theory (Rotter, 1966), this study found that locus of control may have an influence on employees’ proactive behavior. Compared with employees with external locus of control who attribute the reason for failure or success to the external environment, the employees with internal locus of control who believe in their own efforts are more willing to take proactive behavior. Moreover, employees with internal locus of control are more likely to achieve creativity through active learning and task crafting than employees with external locus of control given their power to think actively and make changes.

### Theoretical Implications

Our study demonstrates various theoretical implications. First, the results corroborate the positive association of AIRA with employee creativity, which extends the current research about AIRA. Previous research concerning the outcomes of AIRA has mainly concentrated on negative aspects, such as turnover intention, psychological problems, and working efficiency, to name a few (Brougham and Haar, 2017; Li et al., 2019). The positive influence of AIRA has generally been left unexplored. Our study addresses this research gap and supports the relevant research. Although using AI and robotics causes a threat to employees, they can take a proactive approach to cope with it instead of negatively accepting it. The predicament also predicts an opportunity that employees can improve themselves to become more competitive. In the process of strengthening, employees gain new knowledge, skills, and active thinking process, and ultimately they will gain creativity. Hence, our study also extends the understanding of creativity theory that predicament can serve as an important antecedent variable of creativity.

Second, our study reveals that AIRA is effective in promoting employee creativity *via* driving their active learning and task crafting. This finding provides support for the TWA’s perspective in which employees will tend to take active actions (i.e., active learning and task crafting) to achieve a person-environment fit, which in turn lead to positive outcomes (Caldwell and Iii, 1990; Lee et al., 2021). That is, the usage of AI and robotics in the hotel industry is likely to cause a mismatch between employees and their job. Employees who have AIRA may try to return to the fitting state through active learning and task crafting. Only in the state of fitting job can employees win back their control over their jobs and avoid the negative outcome, such as dismissing or psychological problems caused by the mismatch. In addition, the fitting state also can explain the ultimate generation of creativity which has served as an outcome of person-environment fit in the previous literature (Berg et al., 2010).

Third, our study finds a locus of control exerting a moderating effect on the link of AIRA to active learning as well as the link of AIRA to task crafting. In the locus of control theory, Rotter (1966) argued that the locus of control, as a kind of personality, is divided into internal and external ones. Individuals featured by an internal locus of control believe in their ability to address difficulties, and they tend to work hard to fight with the challenge. Conversely, individuals featured by an external locus of control attribute the reason for failure or success to the external environment (Bakker et al., 2012). Therefore, compared with the employees with an external locus of control, employees with an internal locus of control have a larger possibility of active learning and task crafting in order to achieve harmony between their work and themselves. Our results not only provide support for the locus of control theory but also enrich the understanding of how the interplay between individual cognition and personality (i.e., AIRA and locus of control) can predict individual behavior (i.e., active learning and task crafting).

Moreover, this study develops an integrative moderated mediation model. The model provides solid evidence that the extent to which active learning and task crafting, respectively, mediates the relationship between AIRA and employee creativity. In the process of active learning and task crafting, internal ones are more likely to think actively and generate new ideas than external ones. Internal locus of control will strengthen the mediated effects of active learning and task crafting.

Finally, this study broadens the research of AIRA in the Chinese context. Although the application of AI and robotics in Chinese hotels have become popular just in recent years, the relevant research, especially the study exploring employees’ response to AI and robotics, remains scarce. This study offers valuable insight on how AIRA leads to employee creativity and when this relationship will become stronger. Conducting such research in the Chinese context and enriching the relevant research on AIRA with Chinese empirical evidence is meaningful.

### Practical Implications

Our findings present some implications for practice in hotels. First, our findings have shown that employees can respond positively to the use of AI and robotics and the positive reaction may lead to creativity. This result ought to serve as a guideline to organizations that using AI and robotics is not entirely a bad thing for employees. For organizations, before equipping with AI and robotics, they should first state the potential changes brought by AI and robotics and empower employees to reshape their current tasks in proper ways to cooperate with AI and robotics. Moreover, training programs should be implemented to help employees upgrade themselves. Finally, creating a shared learning atmosphere, forming a culture of trust, freedom, and fairness, and encouraging the interactions between employees are also conducive to motivating active learning and task crafting. For individuals, employees need to understand the potential detriment if they take passive coping ways, which may encourage them to strengthen themselves to win a better future. Additionally, employees should nurture learning a habit and must be flexible to cope with task problems. By conducting task crafting and active learning, employees can overcome job mismatches caused by the emergence of AI and robotics.

Second, we found that internal locus of control strengthens the effects of active learning and task crafting. Hence, organizations need to consider employees’ personalities into consideration. If the organization aims to use or has already used AI and robotics in daily work, the recruitment should concentrate on employees with an internal locus of control who can adapt to changes and be more willing to take proactive behavior. Concerning the existing staff, organizations need to pay attention to the external ones who have great value for the organization. Informing the external ones of their significance to the organization and encouraging them to accept and adapt to the application of AI and robotics are necessary.

### Limitations and Future Research

First, as the data were collected from hotel employees, the range of application of empirical results is limited in practice. For one, our findings are probably not applicable to other industries. With the popularization and application of AI and robotics in other industries, the relevant research will further broaden and deepen. For another, the notion of AIRA is a part of STARA awareness, which limits the richness of this study under the context of the advanced Fourth Industrial Revolution. Therefore, future research can concentrate on STARA and a broader range of industries.

Second, this study adopts the self-reported method to measure employee creativity, which may lead to social expectation deviation. As self-reported creativity is more similar to creative self-efficacy (Ng and Feldman, 2012), it is a relatively subjective concept. Therefore, future studies should measure employee creativity through a more objective method, such as adopting the superior’s evaluation of employee creativity or using other objective evaluation indicators.

Third, this study explores the moderating effect of locus of control on the indirect correlation of AIRA and employee creativity *via* active learning and task crafting. However, as employees could overcome the threat brought by the usage of AI and robotics through active learning and task crafting, these two proactive behaviors may be constrained by the leadership style. For example, authoritarian leadership demanded complete obedience from subordinates, which may restrict employees from reshaping their task content and learning new skills. Thus, future research should also consider leadership style.

## Data Availability Statement

The raw data supporting the conclusions of this article will be made available by the authors, without undue reservation.

## Ethics Statement

The studies involving human participants were reviewed and approved by an institutional review board at the Xiangtan University of China. The participants provided their written informed consent to participate in this study.

## Author Contributions

HW developed the theoretical model and wrote the manuscript. HZ analyzed the data and participated in manuscript writing. ZC, JZ, and YZ collected the data. All authors contributed to the article and approved the submitted version.

## Conflict of Interest

The authors declare that the research was conducted in the absence of any commercial or financial relationships that could be construed as a potential conflict of interest.

## Publisher’s Note

All claims expressed in this article are solely those of the authors and do not necessarily represent those of their affiliated organizations, or those of the publisher, the editors and the reviewers. Any product that may be evaluated in this article, or claim that may be made by its manufacturer, is not guaranteed or endorsed by the publisher.
